# The regulatory role of cancer stem cell marker gene CXCR4 in the growth and metastasis of gastric cancer

**DOI:** 10.1038/s41698-023-00436-2

**Published:** 2023-09-07

**Authors:** Hongying Zhao, Rongke Jiang, Chunmei Zhang, Zhijing Feng, Xue Wang

**Affiliations:** 1 Department of Oncology, Xuzhou City Cancer Hospital, Xuzhou Third People’s Hospital, Xuzhou Hospital Affiliated to Jiangsu University, Xuzhou, 221000 PR China; 2 Jiangsu University, Zhenjiang, 212013 PR China

**Keywords:** Cancer, Cancer

## Abstract

Single-cell RNA sequencing (scRNA-seq) and bulk RNA sequencing (bulk RNA-seq) are increasingly used for screening genes involved in carcinogenesis due to their capacity for dissecting cellular heterogeneity. This study aims to reveal the molecular mechanism of the cancer stem cells (CSCs) marker gene CXCR4 in gastric cancer (GC) growth and metastasis through scRNA-seq combined with bulk RNA-seq. GC-related scRNA-seq data were downloaded from the GEO database, followed by UMAP cluster analysis. Non-malignant cells were excluded by the K-means algorithm. Bulk RNA-seq data and clinical sample information were downloaded from the UCSC Xena database. GO and KEGG pathway analyses validated the correlation between genes and pathways. In vitro and in vivo functional assays were used to examine the effect of perturbed CXCR4 on malignant phenotypes, tumorigenesis, and liver metastasis. A large number of highly variable genes were identified in GC tissue samples. The top 20 principal components were selected, and the cells were clustered into 6 cell types. The C4 cell cluster from malignant epithelial cells might be CSCs. CXCR4 was singled out as a marker gene of CSCs. GC patients with high CXCR4 expression had poor survival. Knockdown of CXCR4 inhibited the malignant phenotypes of CSCs in vitro and curtailed tumorigenesis and liver metastasis in nude mice. CSC marker gene CXCR4 may be a key gene facilitating malignant phenotypes of CSCs, which thus promotes tumor growth and liver metastasis of GC.

## Introduction

Gastric cancer (GC) is highly heterogeneous, molecularly, and phenotypically^1–3^. GC manifests rapid development, distant metastasis, and chemotherapy resistance, in which its tumor heterogeneity exerts an important function^3,4^. Cancer stem cells (CSCs) are tumor cell subpopulations that can induce tumor initiation and lead to relapses^5^, and characterization of CSCs is important for cancer diagnosis, treatment, and prognosis^6^. The participation of gastric CSCs has been suggested to affect the metastasis and recurrence of GC^7^. Indeed, CSCs play unique roles in cancer initiation, progression, metastasis, and chemotherapy resistance^8^. CSCs could generate new tumors by self-renewal and producing differentiated cancer progeny, thereby re-initiating the tumorigenic process. In addition, CSCs are highly invasive and have metastatic potential, mediating epithelial-mesenchymal transition (EMT). In the CSC microenvironment, cytokines such as TGFβ secreted by immune cells such as MSCs, CAFs, TAMs, and MDSCs play important roles in EMT-mediated CSC invasion^9^. CSCs could also mediate chemotherapy resistance, leading to treatment failure and decreased survival rates for cancer patients^10^. The mechanism is related to the deactivation of compounds by overexpression of ATP-binding cassette (ABC) transporters and detoxifying enzymes such as aldehyde dehydrogenases (ALDHs)^11,12^ or through the overexpression of survival-promoting factor BCL2 to resist apoptosis^13^, as well as by efficient DNA repair mechanisms and slower cell division or dormancy rates that result in drug resistance^14,15^.

Heterogeneity in cell populations poses a significant challenge in understanding complex cell biological processes. The analysis of cells at the single-cell level, especially single-cell RNA sequencing (scRNA-seq), has enabled comprehensive dissection of cellular heterogeneity^16^. scRNA-seq is becoming an important strategy to detect cellular transcriptional activity^17^. Interestingly, scRNA-seq can be applied in analysis for GC, such as revealing active cell subtypes and their collaboration in tumor microenvironments^18^. Furthermore, averaging artifacts related to traditional bulk RNA-seq data can be avoided by scRNA-seq^19^. Finally, it should be noted that the scRNA-seq, in combination with bulk RNA-seq performed in the current study, screened C-X-C chemokine receptor type 4 (CXCR4) as a key gene in the growth and metastasis of GC. CXCR4 is identified as a chemokine receptor that is involved in multiple pathological conditions such as immune diseases and cancer^20^. In addition, CXCR4 is one of the CSC marker genes^21^.

Interestingly, the mRNA level of CXCR4 could be increased by cancer stem-like cells^22^. Increased CXCR4 expression by DC-SIGNR was previously reported to augment the liver metastasis of GC^23^. The activated CXCR4 signaling by collagen triple helix repeat containing 1 promoted GC metastasis, involved with the regulation of HIF-1α^24^. CXCR4 expression was also unfolded to share a correlation with the worse prognosis of GC patients and function as a promising prognostic biomarker^25^. Taking the aforementioned reports into account, we thus intend to explore the possible involvement of CXCR4 in GC using scRNA-seq combined with bulk RNA-seq to identify potential genes for treating GC.

## Results

### Analysis of scRNA-seq data identified a large number of highly variable genes in GC tissues

First, seven GC-related scRNA-seq data from GSE163558, including three in situ tumor samples, two lymph node metastasis tumor samples, and two liver metastatic tumor samples, were downloaded through the GEO database, with the R package “Seurat” for scRNA-seq data analysis. Then, after quality control and standardization of the scRNA-seq data, low-quality cells were filtered out (filtering threshold: nFeature_RNA > 500 & nCount_RNA > 1000 & nCount_RNA < 20,000 & percent.mt <10) (Supplementary Fig. 1A). The correlation coefficient (*r*) between nCount and percent.mt was 0.18, and that between nCount and nFeature was 0.91 (Supplementary Fig. 1B), which indicated that the filtered cell quality was good.

Then, highly variable genes in GC were identified from the filtered cells, and a total of 24,924 genes were included for gene expression variance analysis to identify the highly variable genes. The top 2000 highly variable genes regarding variance were finally selected for downstream analysis (Supplementary Fig. 1C).

### PCA of scRNA-seq data of GC tissue samples

Next, we further performed a dimensionality reduction analysis of the GC tissue samples. We used the RunPCA function to reduce the PCA dimension of the top 2000 highly variable genes and found no significant batch effect among the 7 tissue samples (Supplementary Fig. 2A). We used the JackStraw program for heuristic resampling testing: a part of the data was randomly replaced (by default, 1%), and then the PCA was re-run to construct a “zero distribution” of feature scores. We identified the “important” PCs with abundant low *p* values. JackStrawPlot function was used to visualize the top 20 principal components, followed by comparing the distribution of each PC relative to the mean distribution. The “important” PCs usually had a small *p*-value (in the solid line above the dotted line), fully reflecting the information of highly variable genes (Supplementary Fig. 2B). Combined use with the ElbowPlot function showed that the inflection point occurred around the 10th PC, and the change gradually reduced after the 20th (Supplementary Fig. 2C). Here, we selectively displayed the major component genes in the first six PCs (Supplementary Fig. 2D) and drew their heatmap (Supplementary Fig. 2E), indicating that these PCs had distinct DEGs distinguished from other PCs.

To conclude, we selected the top 20 PCs for subsequent UMAP analysis.

### Cells in the GC tissues had obvious heterogeneity

**Fig. 1:**
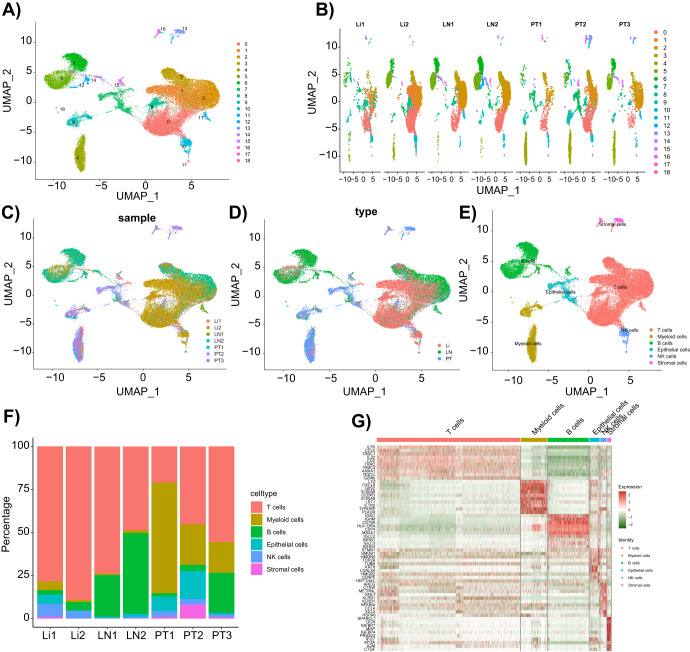
UMAP clustering analysis and cell annotation of scRNA-seq data from GC tissue samples. **A** UMAP clustering analysis grouped cells into 19 cell clusters; **B** UMAP plot shows similarities and differences among cells from different sources; **C**–**D** UMAP plot separately shows similarities and differences among cells from different sources; **E** 19 cell clusters are annotated into 6 cell types; **F** the proportion of each cell type in 7 GC tissue samples; **G** Heatmap of the top 10 marker gene expression in each cell cluster, with boxes of different colors representing differentially expressed genes between different cell types.

Cell heterogeneity in GC tissues was further investigated by UMAP analysis. UMAP combines dimension reduction (such as PCA) with a random walk on the nearest neighbor network to map high-dimensional data to a two-dimensional space while preserving the local distance between cells. Compared with PCA, UMAP is a stochastic algorithm, meaning that multiple methods run on the same dataset will yield different graphs^26^. After analysis by the UMAP method, all the cells were clustered into 19 cell clusters (Fig. 1A). By further correcting the data, we found no significant difference between the different sample sources, but the proportion of the cell clusters was different (Fig. 1B). In addition, we showed the similarities and differences in the cell types based on the data sources (Fig. 1C, D).

**Fig. 2:**
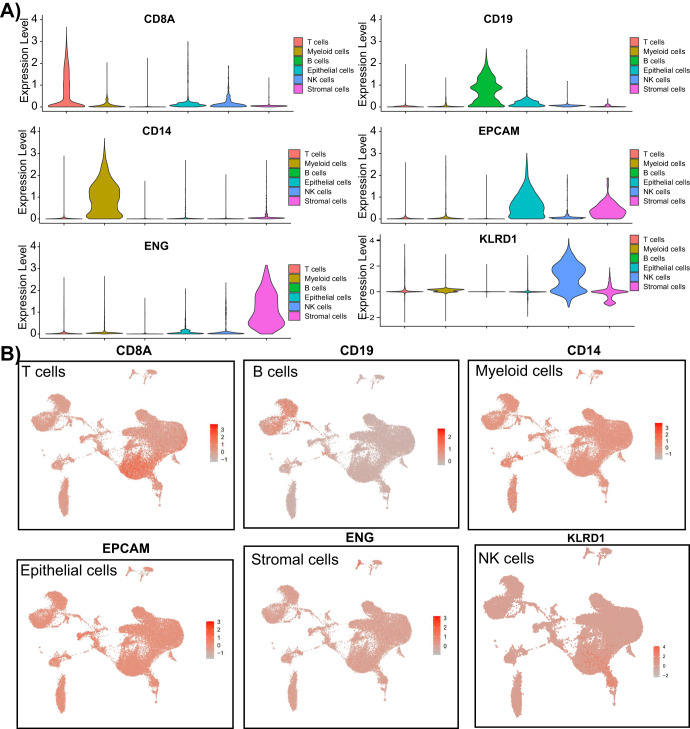
Expression of specific marker genes in different cell types. Violin plots (**A**) and scatter plots (**B**) show the expression of specific marker genes in different cell types.

We annotated 19 cell clusters based on marker genes from the CellMarker database: T cells (CD8A, CD3D, and CD3E), B cells (CD19, CD79A, and MS4A1), Myeloid cells (CD14 and CD163), Epithelial cells (EPCAM, KRT18, and KRT19), NK cells (KLRD1 and IL2RB), and Stromal cells (THY1, ENG, and VWF). Based on these markers, we finally annotated 6 types of cells, with immune cells accounting for 93.8% (Figs. 1E and 2 & Supplementary Table 1). Subsequently, we analyzed the proportion of each type of cell in the GC samples, as shown in Fig. 1F. The proportions of each cell type varied greatly among different GC samples, with epithelial cells, myeloid cells, and stromal cells mainly originating from the primary lesion, while T cells, B cells, and NK cells mainly originating from the metastatic lesion. In addition, we further plotted the expression profile of the top 10 cell-specific marker genes in GC tumor tissue (Fig. 1G).

### GC samples-derived epithelial cells could be further classified into both malignant and non-malignant epithelial cells

**Fig. 3:**
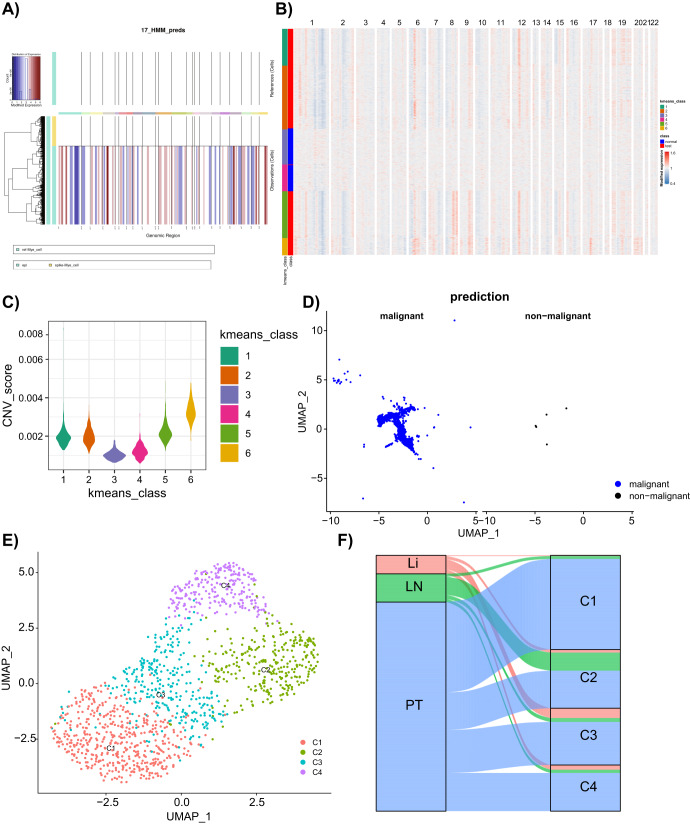
Analysis of epithelial cell malignancy based on the CNV and K-means algorithms. **A** The CNV analysis of the scRNA-seq data. The reference indicates Myeloid cells; yellow in Observation indicates the normal Myeloid cells; green indicates the epithelial cells; blue in the map indicates the loss of a DNA copy; red indicates an increased DNA copy. **B** clustering of the cells of “observation” in Figure A by K-means algorithm. **C** CNV score after K-means clustering. **D** The UMAP plot of the epithelial cells, where blue indicates malignant epithelial cells (1314), and black indicates non-malignant epithelial cells (5). **E** UMAP plot of malignant epithelial cell clustering. **F** Sankey diagram of the distribution of C1–C4 cell clusters in GC tissue samples.

GC originates mainly from the glandular epithelium of the gastric mucosa^27^, and we thus further classified the epithelial cells into both malignant and non-malignant cells. Using Myeloid cells as a control, we used the “inferCNV” package to detect the large-scale cell copy number variation. The results displayed that most of the CNV occurred in the epithelial cells (Fig. 3A). Subsequently, normal and epithelial cells in “Observation” were clustered into six classes, which showed that classes 3 and 4 contained all normal cells with the lowest CNV score, with 1314 malignant epithelial cells subsequently obtained after excluding classes 3 and 4 (Fig. 3B–D).

**Fig. 4:**
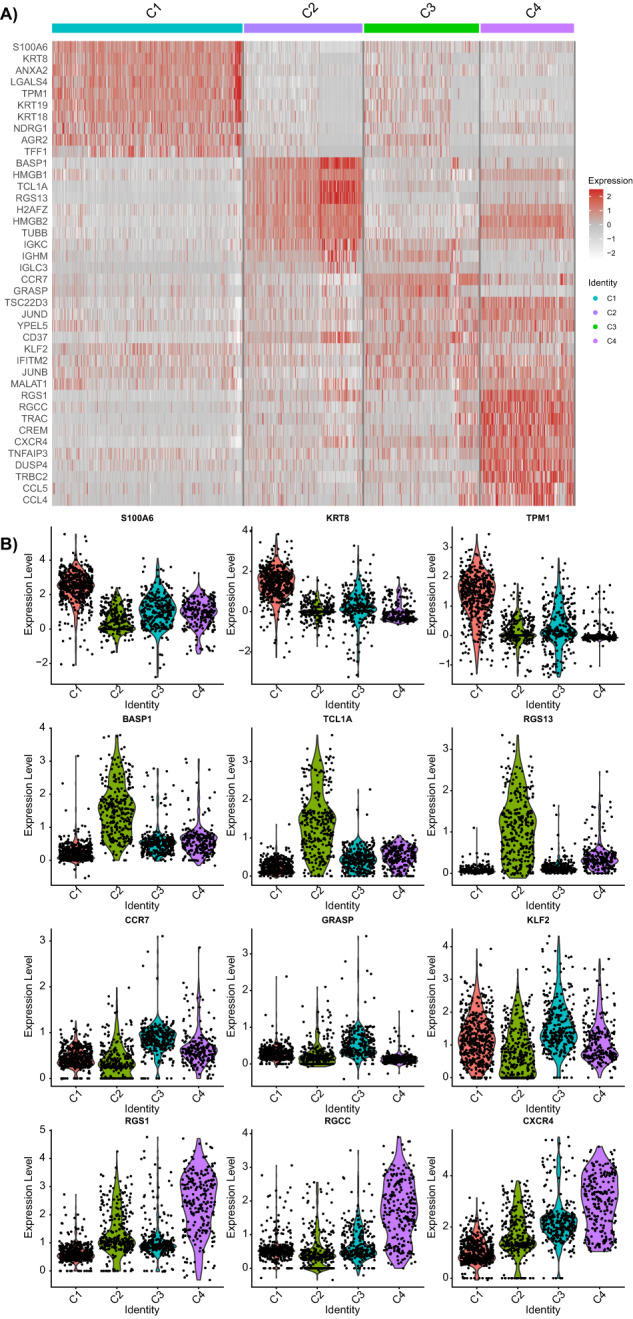
The expression heatmap of the top 10 C1–C4 cell-specific marker genes and violin plot of the expression of three marker genes. The expression heatmap of the top 10 C1–C4 cell-specific marker genes (**A**) and violin plot of the expression of three marker genes (**B**). In the heatmap of Panel **A**, gray represents a low gene expression, while red represents a high gene expression. *p < 0.05, **p < 0.01, ***p < 0.001.

The resulting malignant epithelial cells were further clustered into 4 classes and named C1, C2, C3, and C4, respectively (Fig. 3E). analysis of the source of the C1–C4 GC samples revealed that each GC sample was distributed in the C1–C4 cell cluster (Fig. 3F). Furthermore, we plotted the top 10 cell-specific marker expression profiles genes in clusters C1–C4. We showed the expression of these specific marker genes, namely S100A6, KRT8, and TPM1 in C1, BASP1, TCL1A, and RGS13 in C2, CCR7, KLF2, and GRASP in C3, and RGCC, CXCR4, and RGS1 in C4 (Fig. 4A, B).

### The C4 cell cluster in malignant epithelial cells might be CSCs

**Fig. 5:**
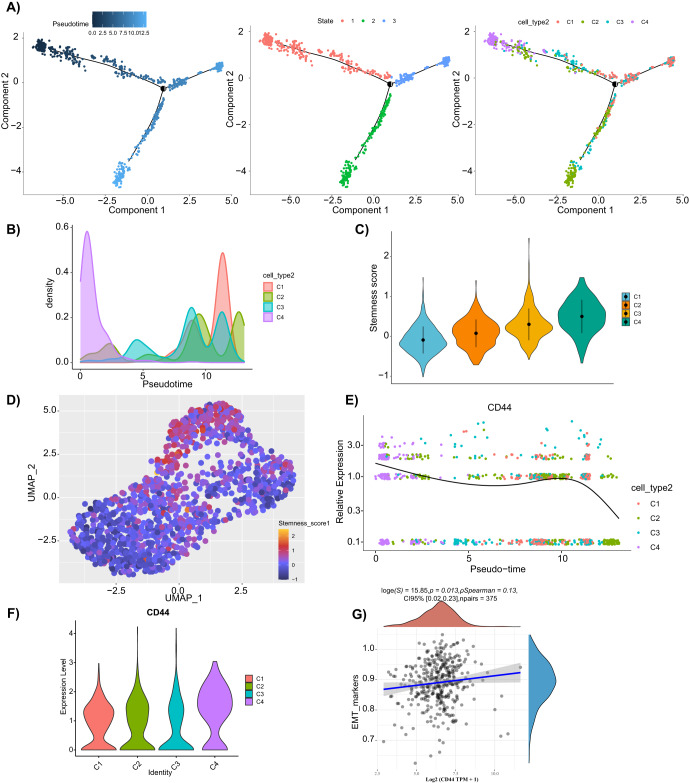
Analysis of CSC clusters in malignant epithelial cells. **A** The Monocle2 analysis of the differentiation trajectory of malignant epithelial cells, presented as pseudotime (left), state (middle), and cell type (right). **B** distribution of the C1–C4 cell clusters on the pseudotime. **C** Stemness score of the C1–C4 cell clusters. **D** UMAP plot of the stemness score of C1–C4 cell clusters. **E** Monocle2 analysis of the expression changes of CD44 with pseudotime in cell clusters C1–C4. **F** Violin plots of CD44 expression in cell clusters C1–C4. **G** The Spearman correlation analysis of CD44 and the EMT marker genes. *p < 0.05, ***p < 0.001.

It has been documented that CSCs may be the key drivers of GC growth and metastasis^28^. Therefore, we first extracted CSCs from malignant epithelial cells. Then, we used the “Monocle2” package for the cell trajectory analysis of the C1–C4 malignant epithelium. Figure 5A, B showed the dynamic characteristics and heterogeneity of the malignant epithelial cells, with the C4 cells located almost entirely at the beginning of the pseudotime trajectory axis. Subsequently, we scored the stemness of C1–C4 cells based on the expression of CSCs markers (TFRC, CXCR4, and JAG1, etc.)^6,29,30^. The results showed that the stemness fraction of C4 cells significantly differed from that of C1–C3 cells (Fig. 5C, D).

In addition, we separately analyzed the expression of the gastric CSCs marker CD44 in the C1–C4 cell clusters and showed that C4 cell clusters had more highly expressed CD44 than the C1–C3 cell clusters (Fig. 5E, F). In addition, cell migration and invasion were strongly correlated with EMT; therefore, we further calculated the correlation between CD44 expression and EMT marker genes according to the ssGSEA algorithm, and the results showed a significant positive correlation (Fig. 5G).

### CSCs marker gene CXCR4 could be used as a molecular marker for prognostic prediction in GC patients

**Fig. 6:**
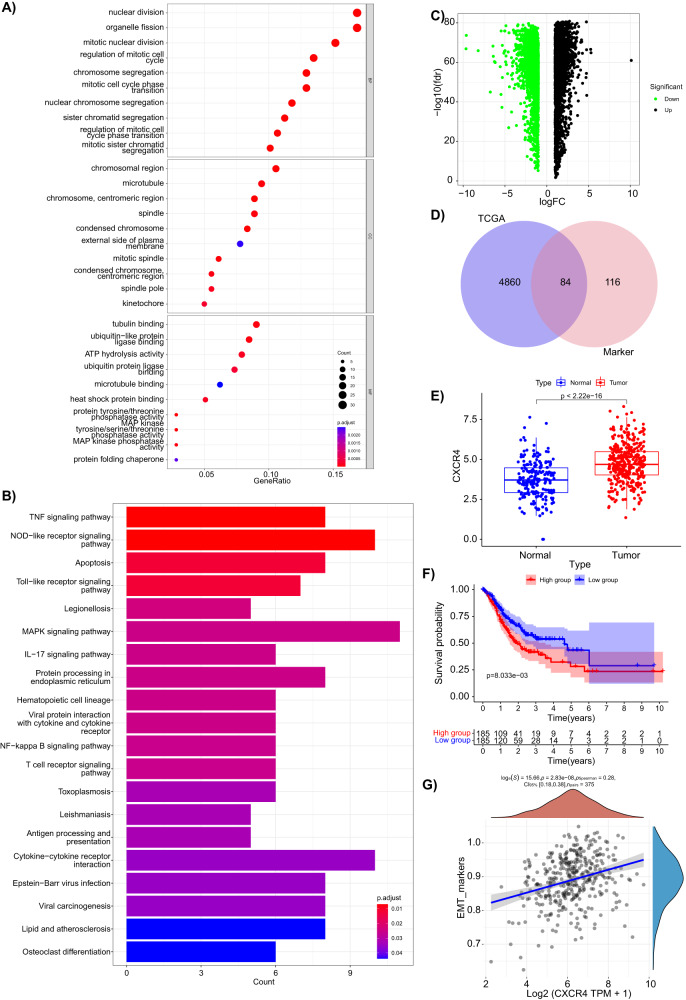
Screening of CSC marker genes associated with prognosis of GC patients and analysis of their relationship with patient prognosis. **A**, **B** Bubble and bar charts of GO and KEGG enrichment analysis of the top 200 marker genes in cluster C4; **C** Volcano plot of differentially expressed genes between GC (*N* = 174 cases) and normal (*N* = 32 cases) samples from TCGA (tumor: *N* = 375 cases, normal: *N* = 174 cases) and GTEx databases; **D** Intersection of differentially expressed genes in GC from TCGA/GTEx and marker genes in cluster C4; **E** Expression of CXCR4 in tumor samples (*N* = 375 cases, red) and normal samples (*N* = 206 cases, black) in GC from TCGA/GTEx databases; **F** Survival curve of patients with high (*N* = 185 cases) and low (*N* = 185 cases) CXCR4 expression in GC; **G** Spearman correlation analysis of CXCR4 gene and EMT marker genes in GC.

Next, the marker genes of CSCs associated with the prognosis of GC patients were further screened. The 200 marker genes of CSCs were subjected to GO and KEGG functional enrichment analyses. The results found that they were mainly involved in biological processes such as nuclear division, organelle fission, mitotic nuclear division, and chromosome segregation, as well as the TNF signaling pathway and NOD-like receptor signaling pathway (Fig. 6A, B).

As shown in Fig. 6C, screening of the DEGs in GC from the TCGA-STAD combined with the GTEx database, these DEGs were then intersected with the 200 marker genes of the C4 cell cluster, which obtained 84 DEGs (Fig. 6D). Further survival prognosis analysis was conducted on these 84 genes, and 10 genes that were significantly associated with survival prognosis were identified, namely RGS2, CXCR4, KIF11, CLSPN, KIF20B, ZNF331, TFRC, UBE2T, CKS2, and EZH2. Among these 10 genes, only CXCR4 showed consistency between the prognosis and expression results, with CXCR4 significantly overexpressed in GC (Fig. 6E) and high CXCR4 expression indicating poor prognosis (Fig. 6F). Therefore, we selected CXCR4 for further study. In addition, the correlation between CXCR4 expression and EMT marker genes was further calculated, and the results showed a significant positive correlation between CXCR4 expression and EMT marker gene expression (Fig. 6G).

### Inhibition of CXCR4 expression suppressed malignant phenotypes of CSCs and retarded tumorigenesis and liver metastasis in vivo

**Fig. 7:**
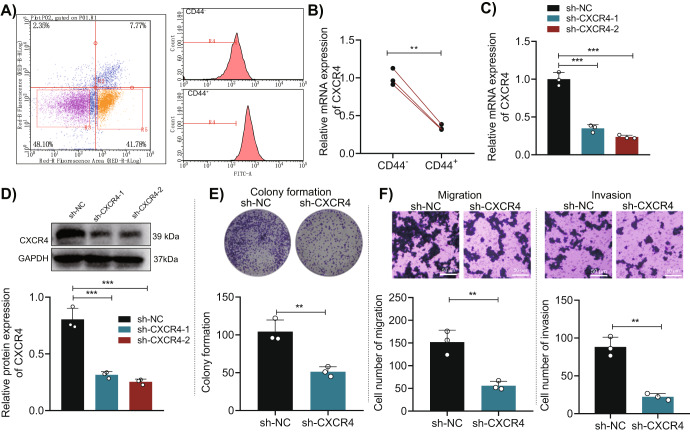
Effects of CXCR4 on proliferation, migration, and invasion of CSCs. **A** Flow cytometry sorted CD44+ and CD44- MKN45 cells; **B** RT-qPCR detected gene expression of CXCR4 in CD44+ and CD44- MKN45 cells; **C** RT-qPCR detected the knockdown efficiency of CXCR4 in CD44 + MKN45 cells; **D** Western blot detected the knockdown efficiency of CXCR4 in CD44 + MKN45 cells; **E** Colony formation assay detected the proliferation ability of each group of CD44 + MKN45 cells; **F** Transwell assay detected the migration and invasion ability of each group of CD44 + MKN45 cells, scale bar = 50 μm. * indicates a significant difference compared to the sh-NC or CD44- group, *p < 0.05, **p < 0.01, ***p < 0.001. Each cell experiment was repeated three times.

First, CD44+ and CD44- MKN45 cells were sorted by flow cytometry (Fig. 7A) and cultured under serum-free conditions. Subsequently, RT-qPCR was performed on the CD44+ and CD44- MKN45 cells, showing that the expression of CXCR4 in CD44 + MKN45 cells was significantly higher than in CD44- MKN45 cells (Fig. 7B).

Then, CXCR4 expression was knocked down in CD44 + MKN45 cells, and the results showed that both sh-CXCR4-1 and sh-CXCR4-2 could significantly reduce the expression of CXCR4 in CD44 + MKN45 cells, with sh-CXCR4-2 (sh-CXCR4) having a higher knockdown efficiency, which was used in subsequent experiments (Fig. 7C, D). Colony formation and Transwell assays showed that compared with the sh-NC group, the proliferation, migration, and invasion abilities of CD44 + MKN45 cells were significantly reduced in the sh-CXCR4 group (Fig. 7E, F).

**Fig. 8:**
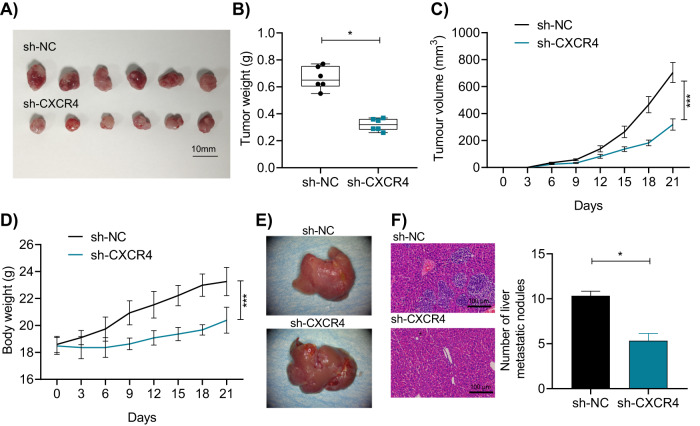
Effects of CXCR4 on in vivo GC tumor formation and liver metastasis. **A** Morphological image of tumor formation experiment in nude mice; **B** Changes in tumor weight in each group of nude mice; **C** Changes in tumor volume in each group of nude mice (0 ~ 9d: *P* > 0.05; 12d: *P* = 0.0128; 15 ~ 21d: *P* < 0.0001); **D** Changes in body weight of each group of nude mice (0 ~ 3d: *P* > 0.05; 6d: *P* = 0.0173; 9 ~ 21d: *P* < 0.0001); **E** Morphology of the liver in each group of nude mice; **F** H&E staining to detect the number of metastatic liver nodules in each group of nude mice, scale bar = 100 μm. * indicates a significant difference compared to the sh-NC group, *p < 0.05, **p < 0.01, ***p < 0.001, with 6 nude mice in each grou*p*.

Next, CD44 + MKN45 cells transfected with sh-NC or sh-CXCR4 were injected subcutaneously or into the tail vein of nude mice to observe tumor growth and liver metastasis. Macroscopically visible tumors were observed in all nude mice, and all survived during the study. The results of tumor growth and weight measurement showed that compared to the sh-NC group, the tumor growth rate in the sh-CXCR4 group of nude mice was significantly reduced, and the tumor weight and volume were significantly smaller, with a significant increase in nude mice weight (Fig. 8A–C). H&E staining was further used to observe the liver metastasis, and the number of metastatic liver nodules was counted. The results showed that compared to the sh-NC group, the number of metastatic liver nodules in the sh-CXCR4 group of nude mice was significantly increased (Fig. 8D–F).

## Discussion

GC is a lethal malignancy with disease heterogeneity and poor prognosis^31^. Herein, we set out to reveal the molecular mechanism of CSC marker gene CXCR4 in GC growth and metastasis by scRNA-seq and bulk RNA-seq and demonstrated CXCR4 as an oncogene in spite.

In the first place, scRNA-seq data analysis conducted in our study found many highly variable genes in GC tissues. Furthermore, we revealed that the cells in GC tissue had obvious heterogeneity. Traditional RNA-seq allows the determination of gene expression variations between multiple cell populations through differential analysis but fails to discover genes contributing to cell-to-cell differences; scRNA-seq enables the determination of highly variable genes within homogeneous cells^32^. scRNA-seq was applied in a previous study, where heterogeneity of tumor cells in GC was dissected^33^. Similarly, prior research using scRNA-seq unfolded the organ-specific metastasis transcriptional heterogeneity in GC, in which stromal cells showed cellular heterogeneity and created a pro-tumoral microenvironment^34^. Bulk RNA-seq of GC tissues was employed to evaluate the tumor microenvironment in GC^35^. Combining scRNA-seq and bulk RNA-seq was also utilized to unveil the heterogeneity of malignant epithelial cells and prognosis signatures in GC^36^. These studies illustrate the feasibility of using scRNA-seq for screening key genes involved in GC progression. In our study, we classified the epithelial cells from GC samples into malignant and non-malignant epithelial cells.

Further, we found that the C4 cell cluster in the malignant epithelial cells might be CSCs. Gastric CSCs can function as fundamental players in the development of GC and lead to heterogeneity of this malignancy^37^. Of note, targeting gastric CSCs has been highlighted to be effective for the therapy of GC, and genes are pivotal regulatory factors in CSCs^38^. Therefore, we further explored important CSC marker genes involved in GC.

Subsequently, we found in the present study that the CSCs marker gene CXCR4 could be used as a molecular marker to predict the prognosis of GC patients and that CXCR4 knockdown suppressed malignant phenotypes of CSCs. Up-regulatedUp-regulated CXCR4 due to HER2 interaction with CD44 diminished miR-139 expression in GC cells, aiding in the promotion of progression and metastasis of GC^39^. It was also revealed that activation of CXCR4 could promote GC metastasis and that overexpressed CXCR4 indicated poor survival of GC patients^40^. Besides, up-regulatedup-regulated CXCR4 displayed unfavorable prognostic significance for GC, and CXCR4 was positively associated with tumor-associated macrophages^41^. To our acknowledgment, the regulation of CXCR4 on gastric CSCs has been increasingly reported. Invasive gastric CSCs were found to be CXCR4 positive and shared a correlation with enhanced metastatic ability^42^. The downregulated CSC marker CXCR4 by MAD2 facilitated stemness and tumorigenesis in GC^43^. Xue et al. used vincristine preconditioning to obtain CSCs from the gastric cancer cell line SGC 7901^22^. These CSCs had self-renewal and differentiation properties, formed 3D structures similar but distinct from the tumor in vitro differentiation assays, and exhibited resistance to multi-drugs and down-regulation of epithelial markers. Fujita et al. found a new marker CXCR4, which induced highly metastatic gastric cancer cells to grow anchorage independently and produce differentiated daughter cells. They were enhanced by using transforming growth factor-β treatment^44^. The results of these studies suggest that identifying and analyzing CSCs in gastric cancer is important for its treatment. These existing reports can support our results about the oncogenic role of CXCR4 in GC. In our study, we provide a more comprehensive view of the transcription profile of primary gastric cancer cells from patients using single-cell RNA-seq and bulk RNA-seq, which helps identify more relevant and diverse cell types present in the tumor microenvironment. Moreover, scRNA-seq can identify heterogeneity in the tumor population and help identify specific cell subgroups, including stem-cell-like cells expressing CXCR4.

**Fig. 9:**
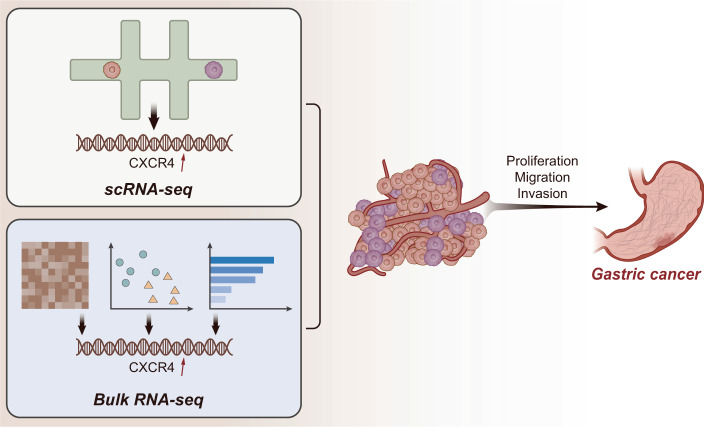
The combination of single-cell RNA sequencing and bulk RNA sequencing analysis reveals the regulatory role of cancer stem cell marker gene CXCR4 in the growth and metastasis of gastric cancer. The cells in GC samples are significantly heterogeneous, and the CSC marker gene CXCR4 may be a key gene enhancing the malignant phenotypes of CSCs, thus promoting GC growth and metastasis.

In conclusion, the results obtained in the current study demonstrated that cells were significantly heterogeneous in GC samples, and the CSC marker gene CXCR4 might be a key gene affecting the growth and metastasis of GC. CXCR4 enhanced the malignant phenotypes of CSCs, thus promoting GC growth and metastasis (Fig. 9). This study may provide a new rationale for screening the CSC marker gene for prognosis, clinical diagnosis, and treatment of GC. Furthermore, CSCs could generate new tumors by self-renewal and producing differentiated cancer progeny, thereby re-initiating the tumorigenic process^8^. CSCs could also generate chemotherapy resistance by deactivating compounds, maintaining a low proliferation rate, resisting apoptosis, and initiating efficient DNA repair mechanisms^10^. Therefore, future studies need to further explore whether CXCR4 mediates gastric cancer occurrence and chemotherapy resistance.

## Methods

### Data collection

We downloaded the single-cell RNA sequencing (scRNA-seq) dataset GSE163558, containing seven gastric cancer (GC) tumor samples (including 3 in situ tumor samples, 2 lymph node metastasis tumor samples, and 2 liver metastasis tumor samples) from the Gene Expression Omnibus (GEO) database (http://www.ncbi.nlm.nih.gov/geo/). We also downloaded the bulk RNA-seq data and clinical information of the GC (TCGA-STAD) cohort as well as RNA-seq data from normal samples of the GTEx database from the UCSC Xena database (https://xenabrowser.net/), which included 375 tumor samples and 32 normal samples in TCGA-STAD, 370 tumor samples in clinical information analysis, and 174 normal samples in GTEx.

### scRNA-seq analysis

We used the Read10X function in the Seurat package (https://CRAN.R-project.org/package=Seurat) to read the raw expression values of the scRNA-seq dataset GSE163558. We then used the CreateSeuratObject function (with parameters min. cells = 3 and min. features = 200) to process the single-cell data and create an object, which retained genes detected in 3 or more cells and cells detecting over 200 genes, automatically calculating the number of genes (nFeature) and RNA molecules (nCount). The PercentageFeatureSet function was used to calculate the mitochondrial genes in cells. Quality control measures were applied to remove potential doublets and low-quality cells. Cells were filtered out if nFeature <200 and nCount <1000 or >20000, while cells with percent.mt >10% were filtered out as well. The FeatureScatter function was used to analyze the correlation between nCount and nFeature.

Then, canonical correlation analysis (CCA) in the Seurat package was applied to remove batch effects. After normalization using the LogNormalize function, highly variable genes were calculated by the FindVariableFeatures function. The RunPCA function was used for principal component analysis (PCA) on the top 2000 highly variable genes. The first 20 PCs were selected for uniform manifold approximation and projection (UMAP) clustering analysis to visualize cellular subtypes using the JackStrawPlot and ElbowPlot functions. CellMarker database (http://xteam.xbio.top/CellMarker/) was used for cell annotation, and marker genes of each cell cluster were obtained using the FindAllMarkers function and the Wilcoxon rank sum test algorithm (with parameters min.pct = 0.3, logFC > 0.25, and FDR < 0.05). Finally, scatter plots and violin plots were generated by the FeaturePlot and VlnPlot functions to visualize gene expression in different cell types.

Subsequently, we extracted the epithelial cells using the subset function, performed CNV analysis on these cells using the inferCNV package (https://bioconductor.org/packages/infercnv/), and used the K-means algorithm to remove non-malignant cells from the epithelial cells. The malignant cells were then analyzed using UMAP clustering. Monocle2 package (https://bioconductor.org/packages/monocle) was used for pseudo-time analysis, data dimensionality reduction using DDRTree, and cell ordering based on gene expression trends. Finally, the AddModuleScore function was utilized for scoring cellular stemness.

### Correlation analysis of the genes and pathways

Genes in the epithelial-mesenchymal transition (EMT) pathway in TCGA were collected. Single-sample gene set enrichment analysis (ssGSEA) was performed using the “GSVA” software package (https://bioconductor.org/packages/GSVA/), and Spearman’s correlation analyzed the correlation between genes and pathway scores.

### Gene functional enrichment analyses

GO and KEGG enrichment analyses of candidate genes were performed with the clusterProfiler package (https://bioconductor.org/packages/clusterProfiler/). The bubble diagram of the enrichment results of biological processes, cell components, and molecular function in GO and the bars of the KEGG enrichment analysis results were drawn using the enrichplot package.

### Differential expression analysis and intersection gene acquisition

Differentially expressed genes (DEGs) in TCGA-STAD were screened by limma package (https://bioconductor.org/packages/limma) in R language with *p* < 0.05 and |logFC | > 1 as the thresholds. Volcano plots of DEGs were drawn using the ggplot2 package (https://CRAN.R-project.org/package=ggplot2) in R language. Marker genes of the C4 cell cluster in scRNA-seq were intersected with the DEGs, and a Venn plot was drawn to obtain key genes.

### Survival analysis

The survival analysis of the target genes was conducted through the “survival” package (https://CRAN.R-project.org/package=survival) in R software.

### Cell culture

Human embryonic kidney cells HEK-293T (Procell, Wuhan, China) and GC cells MKN45 (Procell) were cultured with RPMI-1640 medium (Thermo Fisher Scientific, Rockford, IL) containing 10% FBS and 1% penicillin-streptomycin sulfate (Thermo Fisher Scientific) in an incubator with 5% CO_2_ at 37 °C.

### Lentiviral transduction

The lentiviral vectors harboring short hairpin RNA targeting CXCR4 (sh-CXCR4) or its negative control (sh-NC) were packaged into HEK-293T cells (the final concentration of the shRNA [short hairpin RNA]: 100 nM) using a lentiviral packaging kit (Invitrogen, Carlsbad, CA). The supernatant of the virus was collected after 48 h, and the virus concentration was completed by Genechem (Shanghai, China). Upon about 50% confluence, the MKN45 cells were infected with lentiviral vectors harboring sh-CXCR4 (CXCR4 shRNA) or sh-NC (1 × 10 ^8^ TU/mL) and screened with 10 μg/mL puromycin (Beyotime, Shanghai, China). After 48 h of lentivirus infection, the selection was performed with puromycin, and stable transfection cells was maintained for at least 1 week. The sequences are indicated in Supplementary Table 2.

### RNA extraction and real-time quantitative polymerase chain reaction (RT-qPCR)

According to the manufacturer’s instructions, total RNA was extracted from cells using TRIzol reagent (15596026, Invitrogen, Carlsbad, CA, USA)^45^. The concentration and purity of the extracted total RNA were detected using a Nanodrop 2000 spectrophotometer (1011U, nanodrop, USA, https://www.thermofisher.cn/cn/zh/home.html). Next, 1 µg of total RNA was reverse transcribed into cDNA using the PrimeScriptTM RT reagent kit (RR047A, TaKaRa, Japan). Finally, real-time fluorescent quantitative PCR was performed using the SYBR® Premix Ex Taq II kit (RR820A, TaKaRa, Japan) on the StepOnePlus real-time PCR system (Applied Biosystems). The reaction conditions were: pre-denaturation at 95 °C for 10 min, denaturation at 95 °C for 10 s, annealing at 60 °C for 20 s, and extension at 72 °C for 34 s. There were 40 cycles, and PCR product melting curve analysis was conducted between 65 °C and 95 °C. GAPDH was used as the internal reference primer. The relative transcription level of the target gene was calculated by the 2^-ΔΔCT^ method: ΔΔCt = ΔCt experimental group - ΔCt control group, ΔCt = Ct (target gene) - Ct (internal reference), and the relative transcription level of the target gene mRNA was 2^-ΔΔCt^^46^. The experiment was repeated three times. PCR primers are listed in Supplementary Table 3.

### Western blot

Add RIPA lysis buffer containing PMSF (P0013B, Beyotime, Shanghai, China) to lyse cells and extract total protein. The protein extraction kit (P0028, Beyotime, Shanghai, China) was used for protein extraction according to the instructions. The supernatant was taken, and the total protein concentration of each sample was determined using the BCA assay kit (P0011, Beyotime, Shanghai, China). The protein concentration was adjusted to 1 μg/μL, and each sample volume was set to 100 μL. The samples were boiled at 100 °C for 10 min to denature the proteins and then stored at −80 °C until use.

An 8%–12% SDS gel was prepared based on the target protein band size. Protein samples were loaded onto the gel using a micropipette equal to the volume of each lane and separated by electrophoresis. The separated proteins were transferred onto a PVDF membrane (1620177, BIO-RAD, USA). The membrane was blocked with 5% skim milk or 5% BSA at room temperature for 1 h and then incubated with rabbit anti-GAPDH (5174 S, 1:5000, Cell Signaling Technology, USA) and rabbit anti-CXCR4 (ab181020, 1:1000, Abcam, UK) at 4 °C overnight. The membrane was then washed thrice with 1 × TBST for 5 min at room temperature. Finally, the membrane was incubated with goat anti-rabbit IgG-HRP secondary antibody (ab6721, 1:5000, Abcam, UK) at room temperature for 1 h.

The membrane was washed three times with 1 × TBST buffer for 5 min at room temperature. The membrane was immersed in ECL reaction solution (32109, Thermo Fisher, USA) at room temperature for 1 mi, and then the liquid was removed. The membrane was covered with plastic film and exposed using Image Quant LAS 4000 C gel imaging system (GE, USA) to obtain the band images. GAPDH was used as an internal control for total cellular protein, and the ratio of the grayscale value of the target band to the reference band was used as the relative expression level of the protein^47^. The protein expression level was detected, and the experiment was repeated three times.

### Flow cytometry analysis and fluorescence-activated cell sorting

Approximately 80% of the confluent cells in the cell plate were detached using trypsin-free EDTA and centrifuged at 4 °C. The cell precipitates were re-suspended in HBSS (Gibco, Carlsbad, CA) containing 1 mM HEPES (Gibco) and 2% FBS and filtered with a 40-μm mesh filter. Cells were stained with diluted anti-CD44-FITC (BD Biosciences, Franklin Lakes, NJ) and cultured with 5% CO_2_ at 37 °C for 30 min. Cells were then re-suspended in HBSS containing 1 mM HEPES, 2% FBS, and 1% penicillin-streptomycin sulfate. The MKN45 cells were immediately analyzed and sorted by fluorescence-activated cell sorting using the FACS™ Sample Prep Assistant III (BD Biosciences). The experiment was repeated three times.

### Colony formation assay

200 MKN45 cells were seeded in 24-well plates and cultured in a humidified incubator at 37 °C with 5% CO_2_ for 2 weeks. After that, adherent cells were fixed with 4% paraformaldehyde and stained with 0.5% crystal violet for 10 min. A cell cluster of over 50 cells was considered a colony. The experiment was repeated three times.

### Transwell assay

Transwell assay was performed as previously described^48^. For the migration assay, 5 × 10^4^ MKN45 cells suspended in 200 μL of serum-free medium were seeded in the apical chamber, and the basolateral chamber was supplemented with 800 μL medium containing 20% FBS. After incubation at 37 °C for 16 h, cells were stained with 0.1% crystal violet for 30 min, observed, photographed, and counted under an inverted microscope. For the invasion assay, Matrigel (356234, BD Biosciences; preserved at −80 °C; 50 μL/well) was supplemented to the chamber before the addition of the cells, followed by culture for 24 h. Other steps were the same as those in the migration assay. The experiment was repeated three times.

### Tumor xenograft in nude mouse and construction of liver metastasis model

Twenty-four 5-6-week male immunodeficient nude mice (BALB/c, nu/nu, Vital River, Beijing, China) were separately caged in a specific-pathogen-free animal laboratory (humidity of 60% ~ 65% and temperature of 22–25 °C), with free access to food and water under 12 h light/dark cycles. In addition, the mice were acclimatized for one week before the experiment.

In a subcutaneous tumor model, 1 × 10^6^ CD44^+^ MKN45 cells were injected subcutaneously into the left shoulder of nude mice. In a nude mouse model of liver metastasis, 1 × 10^6^ CD44^+^ MKN45 cells were injected into nude mice via the tail vein. In both models, mice were randomly injected with CD44^+^ MKN45 cells infected with lentiviral vectors harboring sh-NC or sh-CXCR4 (*n* = 6) after sorting CD44^+^ MKN45 cells. After 22 days, all nude mice were sacrificed with CO_2._ Asphyxiation and the tumor and liver tissues were removed for subsequent experiments.

### H&E staining

Hematoxylin and eosin were used for staining the liver tissues^49^. Liver tissue sections were stained with hematoxylin for 5 min, differentiated with 1% ethanol hydrochloride for 3 s, and stained with 5% eosin for about 2 min. Tissue sections were visualized under a microscope.

### Statistical analysis

All data were statistically analyzed using GraphPad Prism 8.0 (GraphPad Software, La Jolla, CA), and all experiments were repeated at least three times. Measurement data are expressed as mean ± standard deviation. Data between the cancer tissues and normal tissues were compared using a paired *t*-test, and an unpaired *t*-test was used to compare the other two groups. Data among multiple groups were compared by one-way ANOVA and those at different time points by two-way ANOVA or repeated measures ANOVA, followed by Tukey’s post hoc tests. Using the log-rank method, the Kaplan-Meier survival curve analysis was performed for statistical tests. *p* < 0.05, *p* < 0.01, and *p* < 0.001 indicated statistically significant differences.

### Reporting summary

Further information on research design is available in the Nature Research Reporting Summary linked to this article.

### Supplementary information

Supplementary Materials

Reporting Summary

## Data Availability

The data supporting this study’s findings are available in the methods and/or supplementary material of this article. Further requests are available from the corresponding author.
