# Supplementary material for: The regulatory role of cancer stem cell marker gene CXCR4 in the growth and metastasis of gastric cancer
Source: NPJ Precis Oncol. 2023 Sep 7;7:86. doi: 10.1038/s41698-023-00436-2 (PMC10484911; doi:10.1038/s41698-023-00436-2)
Supplement: Supplementary file 1 — Supplementary Materials [file 41698_2023_436_MOESM1_ESM.pdf]

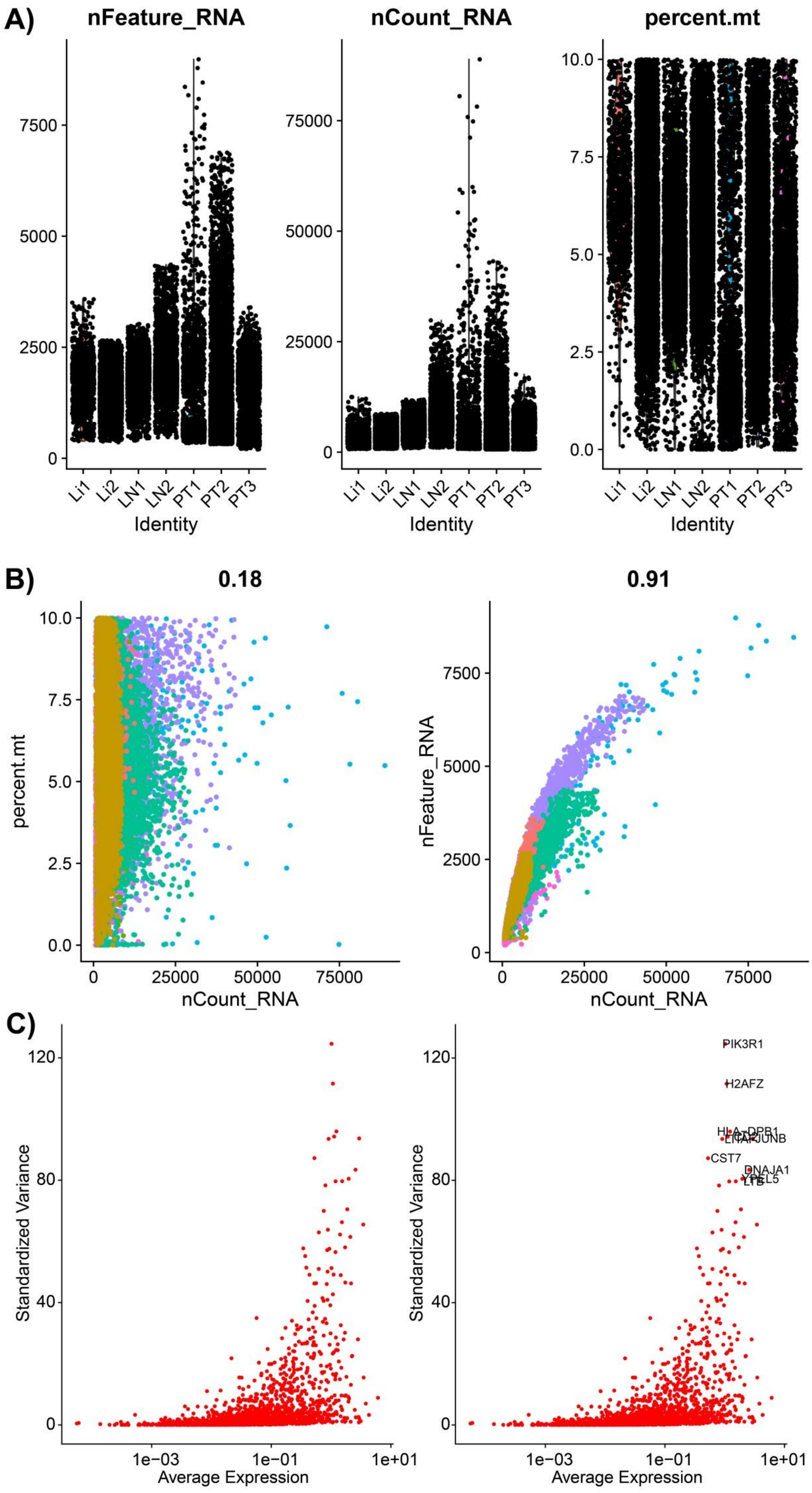

A)
nFeature_RNA
nCount_RNA
percent.mt
Li1
Li2
LN1
LN2
PT1
PT2
PT3
Identity
B)
0.18
0.91
percent.mt
nFeature_RNA
nCount_RNA
C)
Standardized Variance
Average Expression
PIK3R1
H2AFZ
HLA-DPB1
JUNB
CST7
DNAJA1

Supplementary Figure 1 Quality control and variance analysis of single-cell RNA-seq data. A), Quality control was performed on 29,598 cells from 7 GC tissue samples. After quality control, three scatter plots show the quantity of nFeature_RNA, nCount_RNA, and percent.mt in each cell. PT represents the primary tumor, LN represents the lymph node metastatic tumor, and Li represents the metastatic liver tumor; B), Correlation scatter plots show the correlation between nCount and percent.mt (left) and the correlation between nCount and nFeature (right); C), The variable feature plot shows 2,000 highly variable genes selected from the analysis of variance among 21,240 genes.

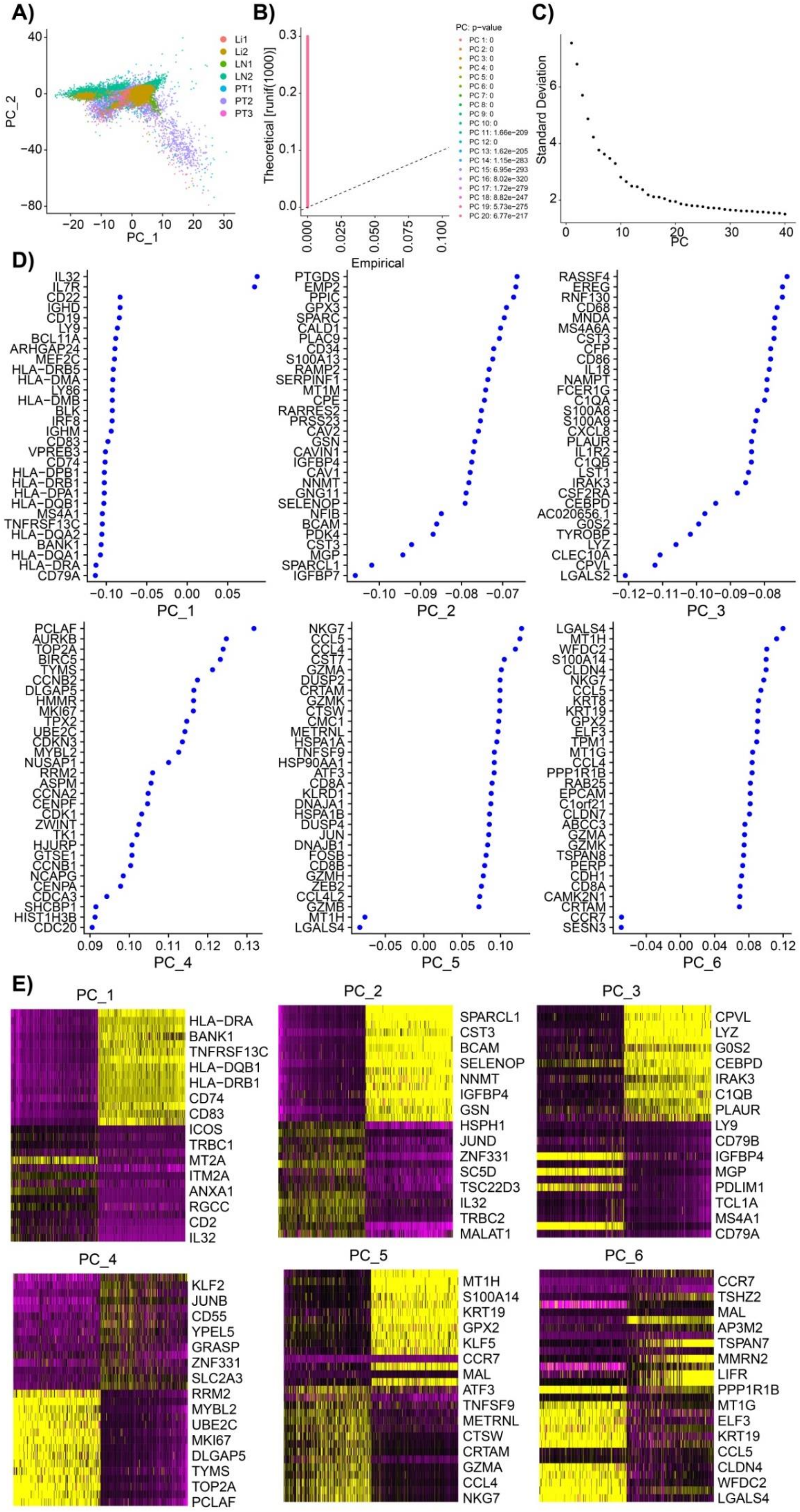
A)
B)
C)
PC_1
PC_2
Li1
Li2
LN1
LN2
PT1
PT2
PT3
Theoretical [runif(1000)]
Empirical
PC: p-value
PC 1: 0
PC 2: 0
PC 3: 0
PC 4: 0
PC 5: 0
PC 6: 0
PC 7: 0
PC 8: 0
PC 9: 0
PC 10: 0
PC 11: 1.66e−209
PC 12: 0
PC 13: 1.62e−205
PC 14: 1.15e−283
PC 15: 6.95e−293
PC 16: 8.02e−320
PC 17: 1.72e−279
PC 18: 8.82e−247
PC 19: 5.73e−275
PC 20: 6.77e−217
Standard Deviation
PC
D)
IL32 IL7R CD22 IGHD CD19 LY9 BCL11A ARHGAP24 MEF2C HLA−DRB5 HLA−DMA LY86 HLA−DMB BLK IRF8 IGHM CD83 VPREB3 CD74 HLA−DPB1 HLA−DRB1 HLA−DPA1 HLA−DQB1 MS4A1 TNFRSF13C HLA−DQA2 BANK1 HLA−DQA1 HLA−DRA CD79A
PC_1
PTGDS EMP2 PPIC GPX3 SPARC CALD1 PLAC9 CD34 S100A13 RAMP2 SERPINF1 MT1M CPE RARRES2 PRSS23 CAV2 GSN CAVIN1 IGFBP4 CAV1 NNMT GNG11 SELENOP NFIB BCAM PDK4 CST3 MGP SPARCL1 IGFBP7
PC_2
RASSF4 EREG RNF130 CD68 MNDA MS4A6A CST3 CFP CD86 IL18 NAMPT FCER1G C1QA S100A8 S100A9 CXCL8 PLAUR IL1R2 C1QB LST1 IRAK3 CSF2RA CEBPD AC020656.1 G0S2 TYROBP LYZ CLEC10A CPVL LGALS2
PC_3
PCLAF AURKB TOP2A BIRC5 TYMS CCNB2 DLGAP5 HMMR MKI67 TPX2 UBE2C CDKN3 MYBL2 NUSAP1 RRM2 ASPM CCNA2 CENPF CDK1 ZWINT TK1 HJURP GTSE1 CCNB1 NCAPG CENPA CDCA3 SHCBP1 HIST1H3B CDC20
PC_4
NKG7 CCL5 CCL4 CST7 GZMA DUSP2 CRTAM GZMK CTSW CMC1 METRNL HSPA1A TNFSF9 HSP90AA1 ATF3 CD8A KLRD1 DNAJA1 HSPA1B DUSP4 JUN DNAJB1 FOSB CD8B GZMH ZEB2 CCL4L2 GZMB MT1H LGALS4
PC_5
LGALS4 MT1H WFDC2 S100A14 CLDN4 NKG7 CCL5 KRT8 KRT19 GPX2 ELF3 TPM1 MT1G CCL4 PPP1R1B RAB25 EPCAM C1orf21 CLDN7 ABCC3 GZMA GZMK TSPAN8 PERP CDH1 CD8A CAMK2N1 CRTAM CCR7 SESN3
PC_6
E)
PC_1
HLA−DRA BANK1 TNFRSF13C HLA−DQB1 HLA−DRB1 CD74 CD83 ICOS TRBC1 MT2A ITM2A ANXA1 RGCC CD2 IL32
PC_2
SPARCL1 CST3 BCAM SELENOP NNMT IGFBP4 GSN HSPH1 JUND ZNF331 SC5D TSC22D3 IL32 TRBC2 MALAT1
PC_3
CPVL LYZ G0S2 CEBPD IRAK3 C1QB PLAUR LY9 CD79B IGFBP4 MGP PDLIM1 TCL1A MS4A1 CD79A
PC_4
KLF2 JUNB CD55 YPEL5 GRASP ZNF331 SLC2A3 RRM2 MYBL2 UBE2C MKI67 DLGAP5 TYMS TOP2A PCLAF
PC_5
MT1H S100A14 KRT19 GPX2 KLF5 CCR7 MAL ATF3 TNFSF9 METRNL CTSW CRTAM GZMA CCL4 NKG7
PC_6
CCR7 TSHZ2 MAL AP3M2 TSPAN7 MMRN2 LIFR PPP1R1B MT1G ELF3 KRT19 CCL5 CLDN4 WFDC2 LGALS4

Supplementary Figure 2 Principal component analysis (PCA) of scRNA-seq data from GC tissue samples. A) PCA analysis shows the clustering of cells from different GC tissues; B), The Jack-Straw plot shows the p-values for the first 20 PCs used for UMAP clustering analysis; C), Elbow plot analysis determines the 20 PCs used for UMAP clustering analysis; D), Scatter plot of genes comprising the top 6 PCs; E), Heatmap of gene expression from the top 6 PCs, where yellow represents high expression in each two-dimensional PC, and purple represents low expression.

Supplementary Table 1 Number of cells in each cell cluster by UMAP cluster analysis

Cell type	Number
T cells	18337
B cells	5290
Myeloid cells	3350
Epithelial cells	1319
NK cells	802
Stromal cells	500
	29598

Note: NK, natural killer

Supplementary Table 2 shRNA sequences

shRNA	Sequence
sh-CXCR4-1	AGATAACTACACCGAGGAAAT
sh-CXCR4-2	CCTGTTCTTAAGACGTGATTT
sh-NC	GCAACAAGATGAAGAGCACCAA

Note: sh-, shRNA, short hairpin RNA; CXCR4, C-X-C chemokine receptor type 4; NC, negative control

Supplementary Table 3 Primer sequences

Gene	Primer sequences (5'-3')
CXCR4	Forward: 5'-CGTCTCAGTGCCCTTTTGTTC-3'
	Reverse: 5'-CTGAAGTAGTGGGCTAAGGGC-3'
GAPDH	Forward: 5'-AGAAGGCTGGGGCTCATTTG-3'
	Reverse: 5'-AGGGGCCATCCACAGTCTTC-3'

Note: C-X-C chemokine receptor type 4; GAPDH, glyceraldehyde 3-phosphate dehydrogenase

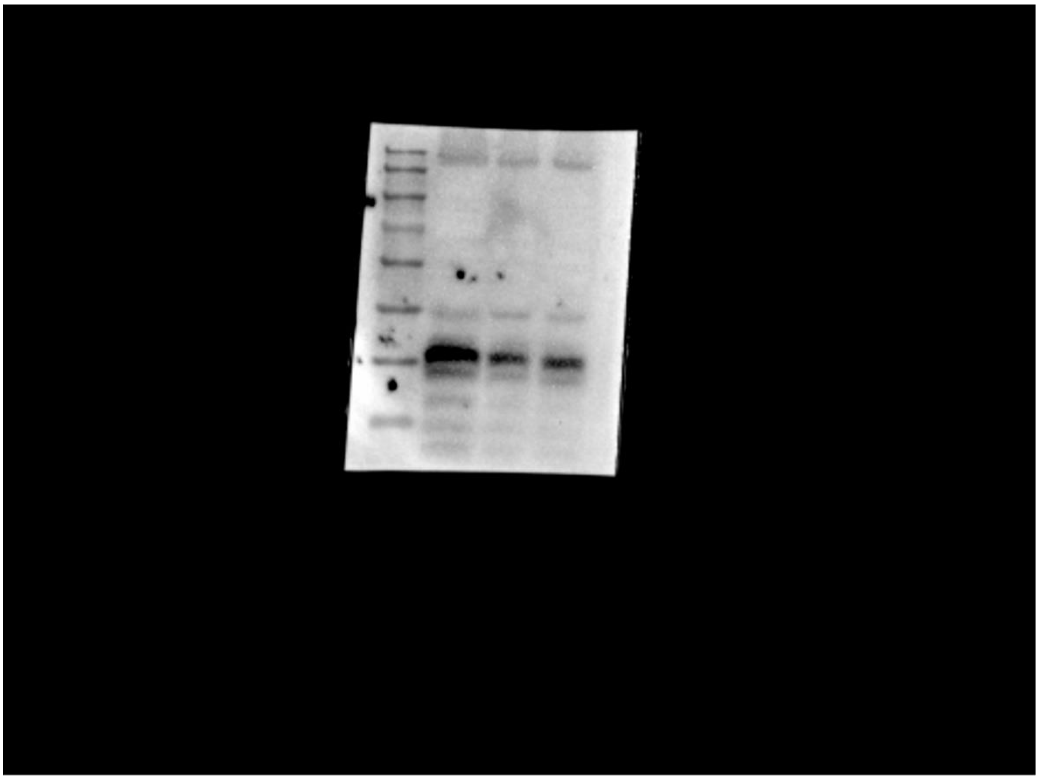

Uncropped scans of figure 7D CXCR4

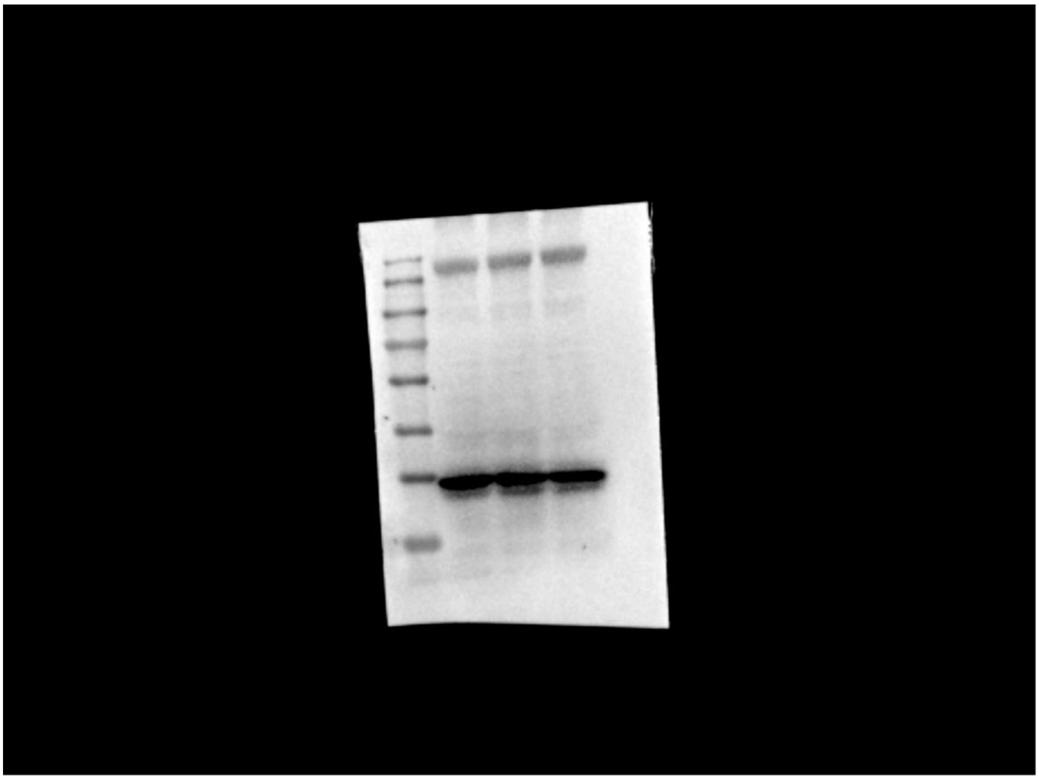,

Uncropped scans of figure 7D GAPDH
