# Supplementary material for: The regulatory role of cancer stem cell marker gene CXCR4 in the growth and metastasis of gastric cancer
Source: NPJ Precis Oncol. 2023 Sep 7;7:86. doi: 10.1038/s41698-023-00436-2 (PMC10484911; doi:10.1038/s41698-023-00436-2)
Supplement: Supplementary file 2 — Reporting Summary [file 41698_2023_436_MOESM2_ESM.pdf]

Corresponding author(s): Hongying Zhao
Last updated by author(s): May 30, 2023

Reporting Summary

Nature Portfolio wishes to improve the reproducibility of the work that we publish. This form provides structure for consistency and transparency in reporting. For further information on Nature Portfolio policies, see our Editorial Policies and the Editorial Policy Checklist.

Statistics

For all statistical analyses, confirm that the following items are present in the figure legend, table legend, main text, or Methods section.

n/a	Confirmed	
☐	☒	The exact sample size (n) for each experimental group/condition, given as a discrete number and unit of measurement
☐	☒	A statement on whether measurements were taken from distinct samples or whether the same sample was measured repeatedly
☐	☒	The statistical test(s) used AND whether they are one- or two-sided<br>*Only common tests should be described solely by name; describe more complex techniques in the Methods section.*
☐	☒	A description of all covariates tested
☐	☒	A description of any assumptions or corrections, such as tests of normality and adjustment for multiple comparisons
☐	☒	A full description of the statistical parameters including central tendency (e.g. means) or other basic estimates (e.g. regression coefficient) AND variation (e.g. standard deviation) or associated estimates of uncertainty (e.g. confidence intervals)
☐	☒	For null hypothesis testing, the test statistic (e.g. F, t, r) with confidence intervals, effect sizes, degrees of freedom and P value noted<br>*Give P values as exact values whenever suitable.*
☐	☒	For Bayesian analysis, information on the choice of priors and Markov chain Monte Carlo settings
☐	☒	For hierarchical and complex designs, identification of the appropriate level for tests and full reporting of outcomes
☐	☒	Estimates of effect sizes (e.g. Cohen's d, Pearson's r), indicating how they were calculated

Our web collection on statistics for biologists contains articles on many of the points above.

Software and code

Policy information about availability of computer code

Data collection	The data supporting this study's findings are available at 10.6084/m9.figshare.23258642
Data analysis	The data supporting this study's findings are available at 10.6084/m9.figshare.23258642

For manuscripts utilizing custom algorithms or software that are central to the research but not yet described in published literature, software must be made available to editors and reviewers. We strongly encourage code deposition in a community repository (e.g. GitHub). See the Nature Portfolio guidelines for submitting code & software for further information.

Data

Policy information about availability of data

All manuscripts must include a data availability statement. This statement should provide the following information, where applicable:

- Accession codes, unique identifiers, or web links for publicly available datasets
- A description of any restrictions on data availability
- For clinical datasets or third party data, please ensure that the statement adheres to our policy

The data supporting this study's findings are available in the methods and/or supplementary material of this article.

Research involving human participants, their data, or biological material

Policy information about studies with human participants or human data. See also policy information about sex, gender (identity/presentation), and sexual orientation and race, ethnicity and racism.

Reporting on sex and gender	Not applicable
Reporting on race, ethnicity, or other socially relevant groupings	Not applicable
Population characteristics	Not applicable
Recruitment	Not applicable
Ethics oversight	Not applicable

Note that full information on the approval of the study protocol must also be provided in the manuscript.

Field-specific reporting

Please select the one below that is the best fit for your research. If you are not sure, read the appropriate sections before making your selection.

☒ Life sciences ☐ Behavioural & social sciences ☐ Ecological, evolutionary & environmental sciences

For a reference copy of the document with all sections, see nature.com/documents/nr-reporting-summary-flat.pdf

Life sciences study design

All studies must disclose on these points even when the disclosure is negative.

Sample size	Not applicable
Data exclusions	Not applicable
Replication	Not applicable
Randomization	Not applicable
Blinding	Not applicable

Reporting for specific materials, systems and methods

We require information from authors about some types of materials, experimental systems and methods used in many studies. Here, indicate whether each material, system or method listed is relevant to your study. If you are not sure if a list item applies to your research, read the appropriate section before selecting a response.

Materials & experimental systems

n/a	Involved in the study	
☐	☒	Antibodies
☐	☒	Eukaryotic cell lines
☒	☐	Palaeontology and archaeology
☐	☒	Animals and other organisms
☒	☐	Clinical data
☒	☐	Dual use research of concern
☒	☐	Plants

Methods

n/a	Involved in the study	
☒	☐	ChIP-seq
☐	☒	Flow cytometry
☒	☐	MRI-based neuroimaging

Antibodies

Antibodies used	CD44-FITC, cat#555478, BD Biosciences
Validation	Flow cytometric analysis of CD44 expression on human peripheral blood lymphocytes. Human whole blood was stained with either FITC Mouse Anti-Human CD44 antibody (Cat. No. 555478/560977; solid line histogram) or FITC Mouse IgG2b, κ Isotype Control (Cat.

No. 555742; dashed line histogram). The erythrocytes were lysed with BD Pharm Lyse™ Lysing Buffer (Cat. No. 555899). The fluorescence histograms were derived from events with the forward and side light-scatter characteristics of viable lymphocytes. Flow cytometric analysis was performed on a BD FACScan™ (BDIS, San Jose, CA).

Eukaryotic cell lines

Policy information about cell lines and Sex and Gender in Research

Cell line source(s)	The MKN45 cells originate from a female patient with gastric adenocarcinoma
Authentication	We authenticated the cells by using short tandem repeat (STR) profiling.
Mycoplasma contamination	We confirmed that the cells were not contaminated by using the RT-qPCR method.
Commonly misidentified lines (See ICLAC register)	Not applicable

Animals and other research organisms

Policy information about studies involving animals; ARRIVE guidelines recommended for reporting animal research, and Sex and Gender in Research

Laboratory animals	Twenty-four 5-6-week male immunodeficient nude mice (BALB/c, nu/nu, Vital River, Beijing, China) were used.
Wild animals	Twenty-four 5-6-week male immunodeficient nude mice (BALB/c, nu/nu, Vital River, Beijing, China) were used in this study. All nude mice were sacrificed with CO2. The tumor and liver tissues were removed for subsequent experiments.
Reporting on sex	Males nude mice have a lower immune response than their female counterparts, leading to a higher rate of tumorigenesis.
Field-collected samples	Nude mice were separately caged in a specific-pathogen-free animal laboratory (humidity of 60% ~ 65% and temperature of 22-25?), with free access to food and water under 12 h light/dark cycles.
Ethics oversight	This study was approved by the Ethics Committee of Xuzhou City Cancer Hospital, and the animal experiments complied with the principles of the management and use of local experimental animals.

Note that full information on the approval of the study protocol must also be provided in the manuscript.

Flow Cytometry

Plots

Confirm that:

- [x] The axis labels state the marker and fluorochrome used (e.g. CD4-FITC).
- [x] The axis scales are clearly visible. Include numbers along axes only for bottom left plot of group (a 'group' is an analysis of identical markers).
- [x] All plots are contour plots with outliers or pseudocolor plots.
- [x] A numerical value for number of cells or percentage (with statistics) is provided.

Methodology

Sample preparation	Approximately 80% of the confluent cells in the cell plate were detached using trypsin-free EDTA and centrifuged at 4°C. The cell precipitates were re-suspended in HBSS (Gibco, Carlsbad, CA) containing 1 mM HEPES (Gibco) and 2% FBS and filtered with a 40-μm mesh filter
Instrument	The MKN45 cells were immediately analyzed and sorted by fluorescence-activated cell sorting using the FACS™ Sample Prep Assistant III (SPA III) (BD Biosciences). The experiment was repeated three times.
Software	The FACS Sample Prep Assistant III is equipped with the BD FACSDiva analysis software, which is used to convert the captured cell types and fluorescent dye information into data.
Cell population abundance	The abundance of CD44+ and CD44- was 40.12% and 46.17%, respectively. The purity of the sample is 89.7%. Purity (%) =(CD44+ cells + CD44- cells) / all cells.
Gating strategy	Dead and live cells were distinguished by labeling with Fixable Viability Dye, whereas the CD44 antibody was used to distinguish CD44+ and CD44- cells.

- [ ] Tick this box to confirm that a figure exemplifying the gating strategy is provided in the Supplementary Information.
